# Segmental analysis of left atrial substrate severity predictors in patients undergoing persistent atrial fibrillation ablation

**DOI:** 10.3389/fcvm.2026.1830208

**Published:** 2026-07-06

**Authors:** Edoardo Cecchini, Gennaro Fabiano, Paola Liporace, Paolo Francesco Sorrenti, Giuseppe Campagna, Emmanuel Fabiano, Jacopo Colella, Alessandro Di Vilio, Giuseppe Indellicati, Simona Brogneri, Diego Sangiorgi, Andrea Petretta, Saverio Iacopino

**Affiliations:** 1 Cardiovascular Department, Maria Cecilia Hospital, GVM Care & Research, Cotignola, Italy; 2 Magna Graecia University of Catanzaro, Catanzaro, Italy

**Keywords:** left atrial substrate, low voltage areas, left atrial wall thickness, epicardial adipose tissue, AF drivers, artificial intelligence, CARTONET

## Abstract

**Introduction:**

Outcomes after catheter ablation remain suboptimal in persistent atrial fibrillation (AF), likely reflecting a more pronounced left atrial (LA) structural and electrical remodeling in these patients. Empirical additional ablation lesions lack robust supporting evidence and may even lead to adverse outcomes, underlining the need to tailor ablation procedures to the individual atrial substrate. The objective of the study was to perform a multidimensional and segment-by-segment assessment of LA substrate anomalies in persistent AF.

**Methods:**

In this single-center retrospective study, 69 consecutive patients undergoing ablation for drug-refractory persistent AF were analyzed. Four factors were selected as possible predictors of LA substrate severity: bipolar voltage, left atrial wall thickness (LAWT), epicardial adipose tissue (EAT) and AF drivers. An AI-based platform was used to segment the LA and to provide detailed analyses.

**Results:**

Among the selected predictors several correlations were identified. Median follow-up duration was 845 days; freedom from recurrence at 12 months was 70.6%. At survival analysis, higher mean percentage of very low-voltage zones (<0.2 mV) predicted recurrences (HR 1.025; *p* = 0.008). Taking the segment location into account, a potentially protective effect of posterior-wall EAT (HR 0.267; *p* = 0.007) and of anterior-wall AF drivers (HR 0.287; *p* = 0.021) was found, while very low bipolar voltage burden in the roof seemed to be associated with an increased risk of recurrence (HR 1.024; *p* = 0.026).

**Discussion:**

The intricate relationship between structural and electrical remodeling in persistent AF underscores the critical role of LA substrate characterization. Understanding the correlations among factors that may indicate atrial substrate abnormalities, including through AI-based platforms, may help deliver a more personalized procedure for each patient.

## 1 Introduction

Pulmonary vein isolation (PVI) is the cornerstone of atrial fibrillation (AF) ablation (1). The outcomes of this treatment are excellent in paroxysmal AF but less satisfactory in persistent forms of the arrhythmia (2, 3), probably reflecting a greater dependence on the left atrial electrical and structural substrate (4). In persistent AF the left atrium (LA) typically exhibits more extensive remodeling, facilitating the development of complex electrical phenomena such as multiple reentries, rotors, and epicardial-endocardial dissociation. To address these mechanisms, patients with persistent AF often undergo additional ablation lines; however, prospective randomized controlled trials have not demonstrated any added benefit in preventing AF recurrence. Furthermore, incomplete linear ablation has the potential to be proarrhythmic, and extensive empirical ablation strategies may have detrimental effects on LA hemodynamic function.

In the absence of clear, definitive evidence regarding the optimal ablation strategy for patients with persistent AF, the need for a more comprehensive characterization of the LA becomes increasingly evident, with the goal to implement a personalized therapeutic approach tailored to each patient’s specific substrate.

The aim of this study was to perform a multidimensional evaluation of LA substrate abnormalities in patients with persistent AF. The LA has been divided into five segments and four factors have been selected as predictors of left atrial substrate severity: bipolar voltage, left atrial wall thickness (LAWT), presence of epicardial adipose tissue (EAT), and AF drivers (regions of interest, ROIs). Interactions between these four atrial substrate severity predictors, their distribution in atrial segments and their impact on post-ablation freedom from recurrence were investigated.

## 2 Methods

In this observational retrospective single-center study a total of 69 consecutive patients who underwent AF ablation for drug-refractory persistent AF at Maria Cecilia Hospital (Cotignola, Italy) were evaluated. Both first procedures and redo cases were included.

The study was approved by the Romagna Ethics Committee on 8 May 2020 (protocol no. CE 3791/2020) and was conducted in full compliance with the principles of the Declaration of Helsinki. Written informed consent was obtained from all patients.

Antiarrhythmic drugs were discontinued for at least five half-lives prior to the procedure. All patients underwent pre-procedural cardiac multi-detector computed tomography (MDCT) the day before the procedure using a 384-slice scanner (SOMATOM Force CT, Siemens Healthineers).

High-density electroanatomic mapping of the LA was performed in AF using a multi-spine catheter (Pentaray, Biosense Webster, Johnson & Johnson MedTech, Irvine, CA, USA).

In ablation-naïve patients and in redo cases where PV reconnection was observed, wide antral circumferential PVI was performed based on the CLOSE protocol (5).

The presence of AF drivers was investigated using CARTOFINDER® (Biosense Webster, Johnson & Johnson MedTech, Irvine, CA, USA), a specialized module within the CARTO system designed to automatically detect focal or rotational activation sites in the LA (6).

Additional LA substrate modification with linear ablation using a customized approach was performed following a tailored assessment based on both the presence of ROIs and voltage substrate findings.

Electrical cardioversion was performed if sinus rhythm was not restored during the procedure.

After achieving sinus rhythm, a new substrate map was performed to confirm PV isolation, and conduction block along linear lesions.

In this study the bipolar voltage data from the electro-anatomical maps, acquired in AF, were analyzed using CARTONET™ release 20 (Biosense Webster, Johnson & Johnson MedTech, Irvine, CA, USA), a cloud-based platform designed to perform AI-assisted automated analysis of previously acquired electro-anatomical maps. This platform allows physicians to securely store, review, and analyze data collected during ablation procedures performed with the CARTO mapping system.

After accessing previously uploaded cases on CARTONET, electroanatomical LA maps were automatically segmented into predefined anatomical regions (anterior wall, posterior wall, inferior wall, roof, septum, lateral wall, PVs, and appendage). In this study PVs, the LA appendage and the interatrial septum were excluded from further analysis. The system then performed automated segment by segment analyses of bipolar voltage maps acquired in AF, providing the results in easily exportable data formats. For each segment the presence of low voltage zones (LVZ, regions with bipolar voltage <0.5 mV and >0.2 mV), very low voltage zones (vLVZ, regions with bipolar voltage <0.2 mV) and the median voltage value were noted.

The presence of ROIs identified by the CARTOFINDER module was also documented. We assessed for each atrial segment whether at least one ROI, focal activity, or rotational activity was present.

For this study, LAWT and EAT were obtained processing MDCT images with the ADAS3D® software (ADAS3D Medical, Barcelona, Spain). LAWT was obtained by calculating, for each endocardial point, the corresponding distance to the epicardial surface. The endocardial surface was delimited with a semi-automated segmentation method guided by intensity thresholds, while the epicardial layer was defined automatically using the software's integrated AI segmentation workflow, and manually corrected where required. LAWT maps were color-coded: red <1 mm, yellow 1–2 mm, green 2–3 mm, blue 3–4 mm, and purple ≥4 mm. For each patient and for each atrial segment we recorded the presence or absence of low thickness zones (<1 mm) or high thickness zones (>4 mm).

Assessing Hounsfield unit density, the ADAS3D® software also identified adipose tissue, thereby displaying EAT deposits on the three-dimensional shell of the left atrium, and providing quantitative evaluations of the volume and area of adipose tissue. We recorded for each patient and for each atrial segment the presence or absence of epicardial fat in that wall.

**Figure 1:**
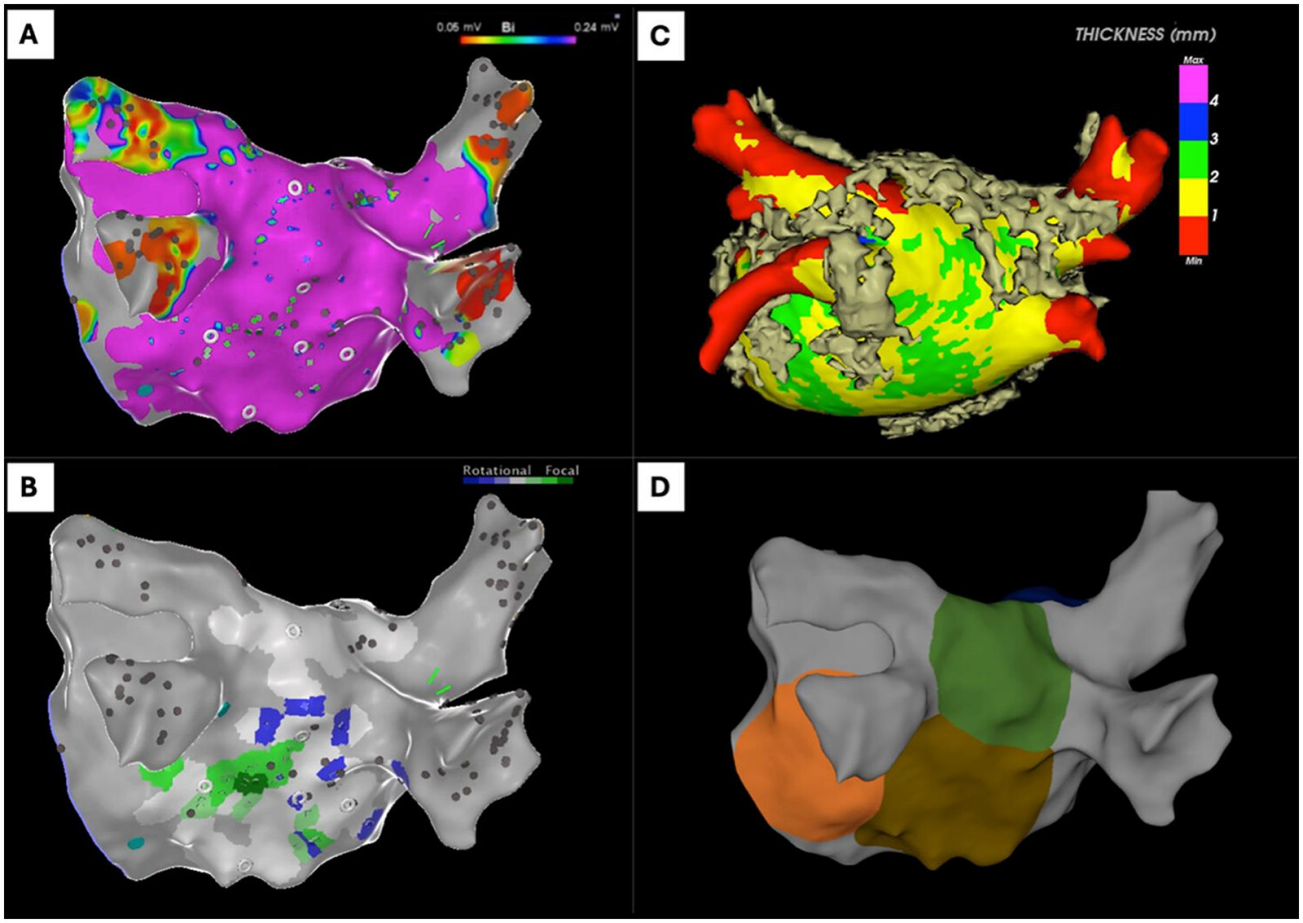
Multidimensional assessment of left atrium. **(A)** Bipolar voltage mapping with CARTO **(B)** CARTOFINDER map **(C)** Pre-procedural multidetector computed tomography processed with ADAS3D showing wall thickness and epicardial adipose tissue **(D)** Segmentation performed with CARTONET.

The allocation of the predictors across the different segments was automated for CARTONET-derived voltage analysis, while for the remaining three variables the allocation was performed manually and simultaneously by the same three operators, all with extensive experience in electroanatomical mapping systems, using as a reference the segmentation automatically generated by CARTONET (Figure 1).

At follow-up, recurrence of atrial arrhythmias was defined as any episode lasting >30 s, after an 8-week blanking period post-ablation.

### 2.1 Statistical analysis

Continuous variables were summarized as mean and standard deviation or median and interquartile range according to normal distribution. Categorical variables were reported as absolute frequencies and percentages. For comparisons across groups, the Kruskal–Wallis test was used, followed by the Dwass–Steel–Critchlow–Fligner *post-hoc* test for non-parametric pairwise comparisons. Comparisons of categorical variables were conducted using the *χ*^2^ test or Fisher's exact test, with adjustment for multiple testing using the Holm correction. Pairwise correlations were quantified using Spearman's rank correlation, Pearson's Phi correlation coefficient or point-biserial correlation according to type of variables. The extent of missing data was evaluated for all variables prior to analysis. Missingness for the covariates included in the model ranged from 29% to 31% across variables; missing data were imputed using random forest imputation for categorical variables and predictive mean matching for continuous variables. The imputation was performed with 100 trees and 5 iterations, using predictive mean matching with k = 3 to ensure realistic values for continuous variables. Time-to-event analyses were carried out using Cox proportional hazards regression. During follow-up, 23 recurrence events were observed. Variable selection was performed using Least Absolute Shrinkage and Selection Operator (LASSO) regularization with 50-fold cross-validation. Candidate variables included redo procedures and substrate characteristics across the five atrial segments (the presence of very low voltage zones, ROIs, epicardial adipose tissue, wall thickness and the application of additional ablative lines in that segment). The proportional hazards assumption was evaluated using Schoenfeld residuals. Model discrimination was assessed using Harrell's concordance index. The risk of overfitting appeared to be limited, as the events-per-variable ratio was close to the commonly accepted 10:1 rule of thumb. Segment-level analyses were performed treating each atrial segment as an independent observational unit. No adjustment for within-patient clustering was applied. All analysis were performed with R 4.5.0 (R Foundation for Statistical Computing, Vienna, Austria); *p*-values < 0.05 were considered statistically significant.

## 3 Results

### 3.1 Baseline characteristics

Baseline characteristics of the study population are summarized in Table 1; procedural aspects are summarized in Table 2.

**Table 1:** Baseline characteristics.

Variable	Total (*n* = 69)
Age, years	64.3 ± 8.5
Male sex, n	51 (74%)
BMI, kg/m^2^	29.1 ± 5.0
BSA, m^2^	2.03 ± 0.21
Hypertension, n	46 (67%)
Hypercholesterolemia, n	26 (38%)
Diabetes Mellitus, n	5 (7%)
Smoking habit, n	47 (69%)
Coronary artery disease, n	3 (4.3%)
Chronic kidney disease, n	4 (6%)
GFR (C–G), mL/min	93.4 ± 25.3
CHA₂DS₂-VASC score	2 (2)
Left atrial area, cm^2^	26.0 (± 4)
LVEF, %	55.8 ± 6.1
Time from first AF episode, months	40 (91)
CIED, n	22 (32%)
Number of AADs tested, n	2 (1)
ACE inhibitors, n	15 (23%)
Calcium channel blockers, n	10 (16%)
Diuretics, n	25 (39%)
Statins, n	20 (31%)
Digoxin, n	8 (13%)
Flecainide, n	14 (22%)
Amiodarone, n	5 (14,7%)
Sotalol, n	10 (29,4%)
Propafenone, n	1 (2.9)
Apixaban, n	8 (23.5%)
Edoxaban, n	5 (14.7%)
Rivaroxaban, n	13 (38.2%)
Dabigatran, n	6 (17.6%)
No anticoagulation, n	2 (5.9%)
Antiplatelet agents, n	3 (4.3%)

BMI, Body Mass Index; BSA, Body Surface Area; GFR (C-G), Glomerular Filtration Rate (Cockcroft-Gault); LVEF, Left Ventricular Ejection Fraction; CIED, Cardiac Implantable Electronic Device; AADs, Anti-Arrhythmic Drugs.

**Table 2:** Procedural aspects.

Variable	Total (*n* = 69)
Redo procedure, n	27 (39%)
Total procedure duration, min	232.6 ± 53.99
Total fluoroscopy time, min	13.9 ± 7
Fast Anatomical Mapping time, min	15.5 ± 5.52
Left atrial volume, mL	158.3 ± 45.79
Number of high-density mapping points, n	5302 ± 2887
Patients undergoing additional ablation beyond PVI, n	56 (81%)
ThermoCool SmartTouch SF ablation catheter, n	34 (50,7%)
QDOT Micro ablation catheter, n	33 (49,3%)

Ablation procedures were performed between April 2021 and November 2023. Overall, 27 (39%) patients had previously undergone ablation procedures: of these, 7 had received cryoablation, and the remaining cases had been treated with radiofrequency, mostly limited to PVI. In two patients, additional ablation lines in the posterior wall had been performed during earlier interventions, and one patient had undergone two prior procedures.

### 3.2 Segmental analysis of left atrial substrate severity predictors distribution

**Figure 2:**
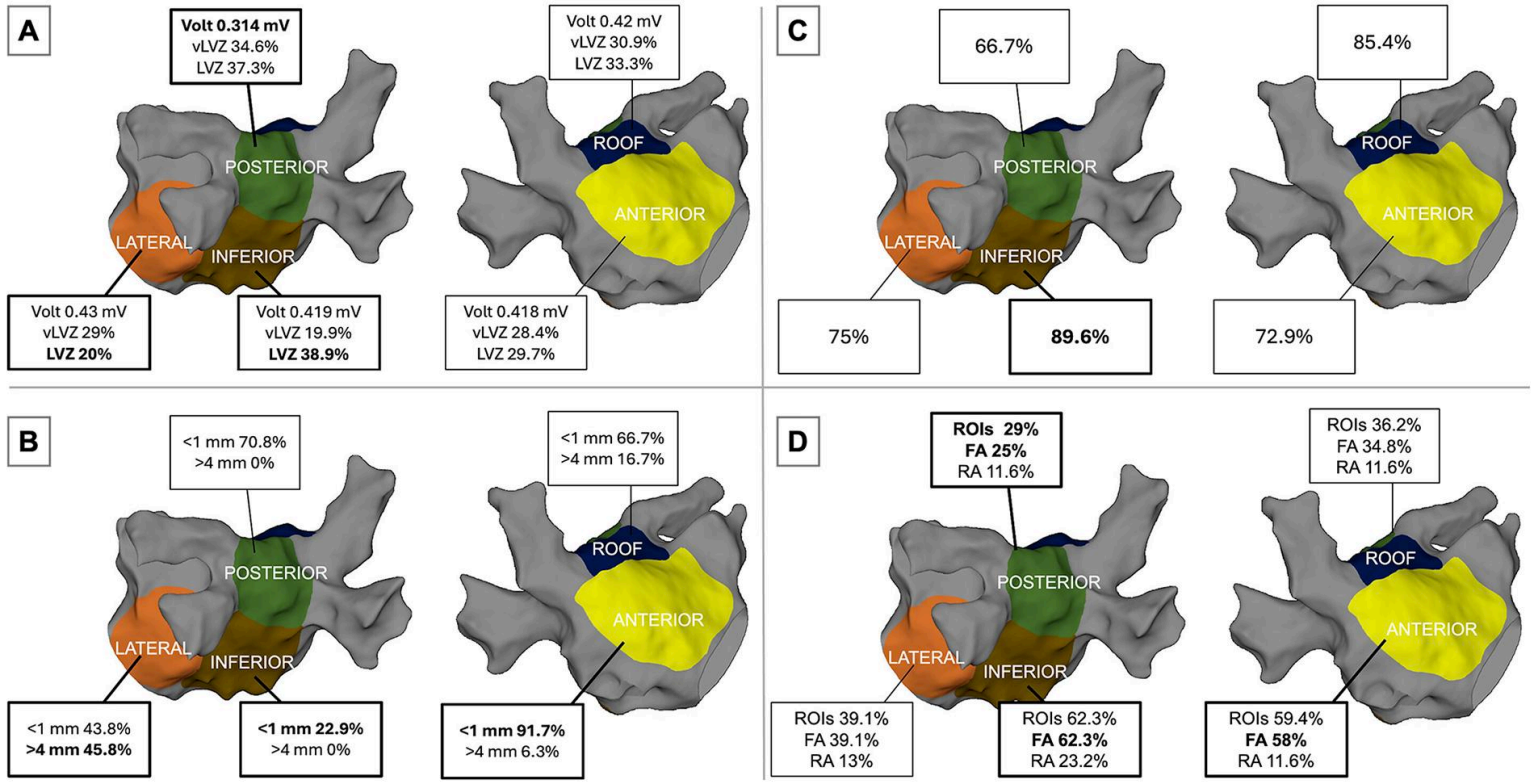
Graphic representation of the distribution of the four predictors across left atrial segments. For each segment is depicted: **(A)** mean value of bipolar voltage (Volt), percentage of vLVZ and of LVZ; **(B)** percentage of patients presenting zones <1 mm or >4 mm thick in that specific LA segment; **(C)** percentage of patients presenting EAT in that specific LA segment; **(D)** percentage of patients presenting ROIs, Focal Activity (FA) and Rotational Activity (RA) in that specific LA segment. Bold values indicate segments that significantly differ from the others.

We evaluated the presence of each predictor across all atrial segments under examination (Tables 3–6, Figure 2).

**Table 3:** Segmental distribution of bipolar voltage: median voltage (V) and percentage of areas with vLVZ and LVZ.

Region	Volt, mV (IQR)	%vLVZ (IQR)	%LVZ (IQR)
GLOBAL LA	0,733 (0,577)	46,5 (16,9)	37,1 (49,8)
POSTERIOR WALL	0,314 (0,320)	34,6 (26,9)	37,3 (20,1)
INFERIOR WALL	0,419 (0,341)	19,9 (21,7)	38,9 (23,6)
LEFT LATERAL WALL	0.43 (0.37)	29 (29)	20 (20)
ANTERIOR WALL	0,418 (0,327)	28,4 (24,1)	29,7 (19,4)
ROOF	0,42 (0.28)	30,9 (29,2)	33,3 (20,3)

**Table 4:** Segmental distribution of LAWT: number and percentage of patients with regions <1 mm or >4 mm.

Region	<1 mm, n (%)	>4 mm, *n* (%)
POSTERIOR WALL	34 (70.8%)	0 (0.0%)
INFERIOR WALL	11 (22.9%)	0 (0.0%)
LEFT LATERAL WALL	21 (43.8%)	22 (45.8%)
ANTERIOR WALL	44 (91.7%)	3 (6.3%)
ROOF	32 (66.7%)	8 (16.7%)

**Table 5:** Segmental distribution of EAT: number and percentage of patients with EAT.

Region	EAT, *n* (%)
POSTERIOR WALL	32 (66.7%)
INFERIOR WALL	43 (89.6%)
LEFT LATERAL WALL	36 (75.0%)
ANTERIOR WALL	35 (72.9%)
ROOF	41 (85.4%)

**Table 6:** Segmental distribution of ROIs: number and percentage of patients with ROIs, FA, and RA.

Region	ROIs, *n* (%)	FA, *n* (%)	RA, *n* (%)
POSTERIOR WALL	20 (29%)	17 (25.0%)	8 (11.6%)
INFERIOR WALL	43 (62.3%)	43 (62.3%)	16 (23.2%)
LEFT LATERAL WALL	27 (39.1%)	27 (39.1%)	9 (13.0%)
ANTERIOR WALL	41 (59.4%)	40 (58.0%)	8 (11.6%)
ROOF	25 (36.2%)	24 (34.8%)	8 (11.6%)

vLVZ, very low voltage zones; LVZ, low voltage zones; LAWT, left atrial wall thickness; EAT, epicardial adipose tissue; ROIs, regions of interest; FA, focal activity; RA, rotational activity.

In the following sections we report the main and most significant findings from our analysis. For the complete results, please refer to the Supplementary Materials.

### 3.3 Correlation between atrial substrate severity predictors and anthropometric - biochemical parameters

A greater presence of EAT was found to be associated with higher BSA (rho = 0.543; *p* = 0.004) and higher BMI (rho = 0.464; *p* = 0.002). Moreover, a trend between EAT and LA dimensions was also observed: the number of segments with EAT correlates with both LA area (rho = 0.643; *p* < 0.001) and LA volume (rho = 0.298; *p* = 0.049). A greater extent of low-voltage areas was found to be associated with a lower glomerular filtration rate (rho = −0.404; *p* = 0.08). Finally, older age was associated with a higher mean percentage of LVZs (*p* = 0.026) and vLVZs (*p* = 0.031).

### 3.4 Correlation between atrial substrate severity predictors

**Figure 3:**
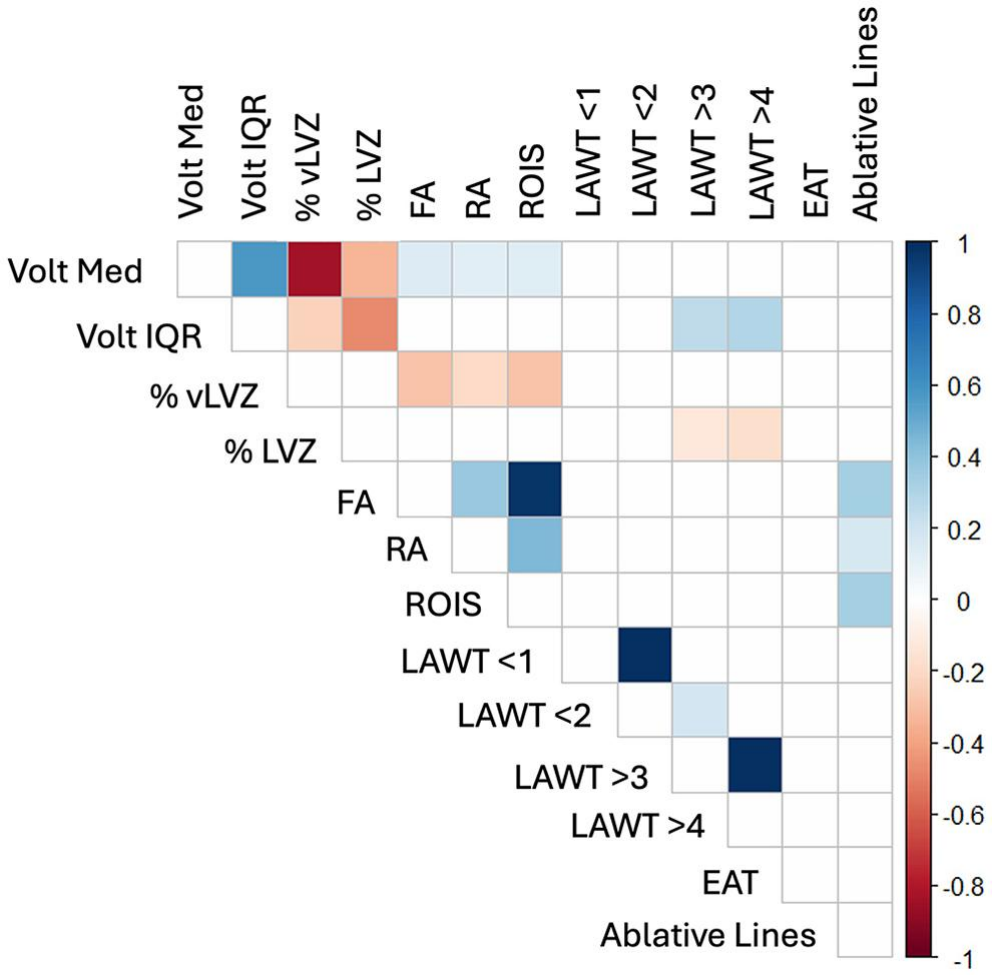
Heatmap of the significant correlations among the severity predictors within each segment. Volt, Voltage; IQR, interquartile range; FA, focal activity; RA, rotational activity.

The correlation among the four factors was evaluated. This analysis was conducted for each segment, without accounting for segment location or the patient to whom the segment belonged, and for each patient, to investigate how the presence or extent of one of the evaluated factors correlated with the presence of each of the others. Figure 3 shows a heatmap of statistically significant correlations within each segment.

In the overall analysis, considering together the results of the segment-level and patient-level association analyses, we identified several significant correlations.

#### 3.4.1 LAWT

**Figure 4:**
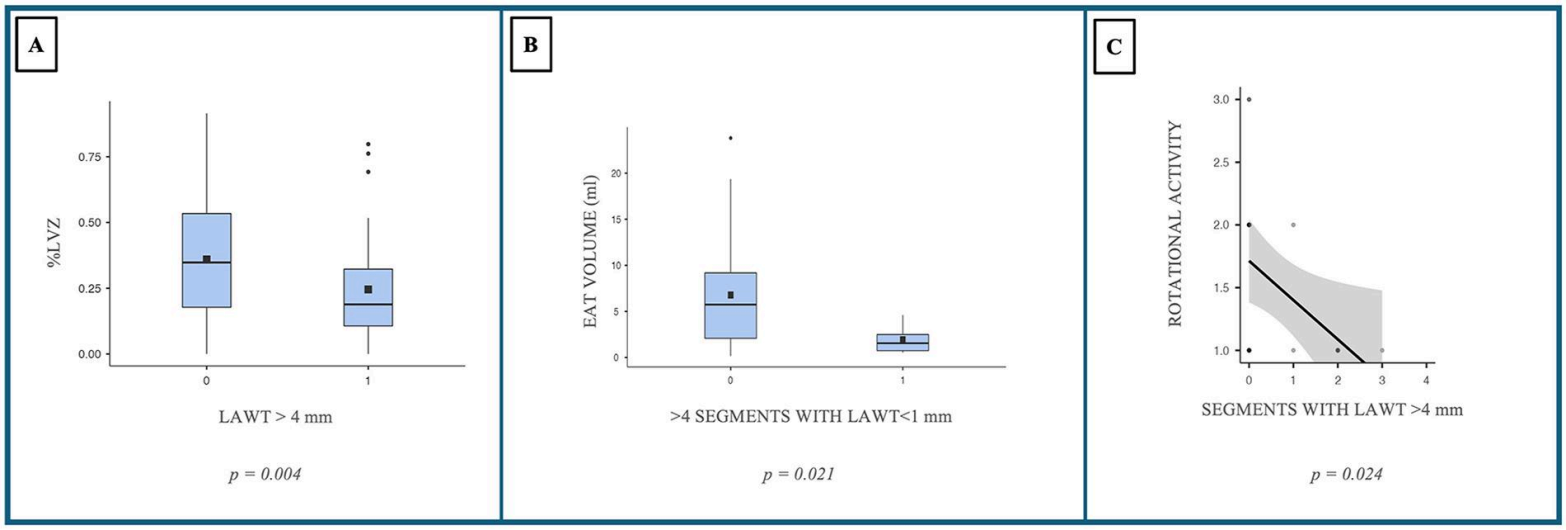
Correlation between wall thickness (LAWT) and: **(A)** percentage of low voltage zones (%LVZ); **(B)** epicardial adipose tissue (EAT) volume; **(C)** rotational activity.

An increase in this parameter has been shown to be associated with higher bipolar voltages, a greater amount of EAT, and a lower prevalence of rotational activity (Figure 4).

Specifically, the presence of areas > 4 mm thick was associated in the same segment with a reduced presence of LVZs (*p* = 0.004), and the presence of at least two segments with thickness >4 mm was associated with a lower prevalence of vLVZs (*p* = 0.023) in the same patient.

The presence of at least four segments with zones of LAWT < 1 mm was significantly associated with a smaller EAT volume and area in the same patient (*p* = 0.021 and *p* = 0.020).

Regarding the relationship between ROIs and LAWT, a trend was observed between a greater wall thickness and fewer rotors (rho = −0.478; *p* = 0.024).

#### 3.4.2 Bipolar voltage

**Figure 5:**
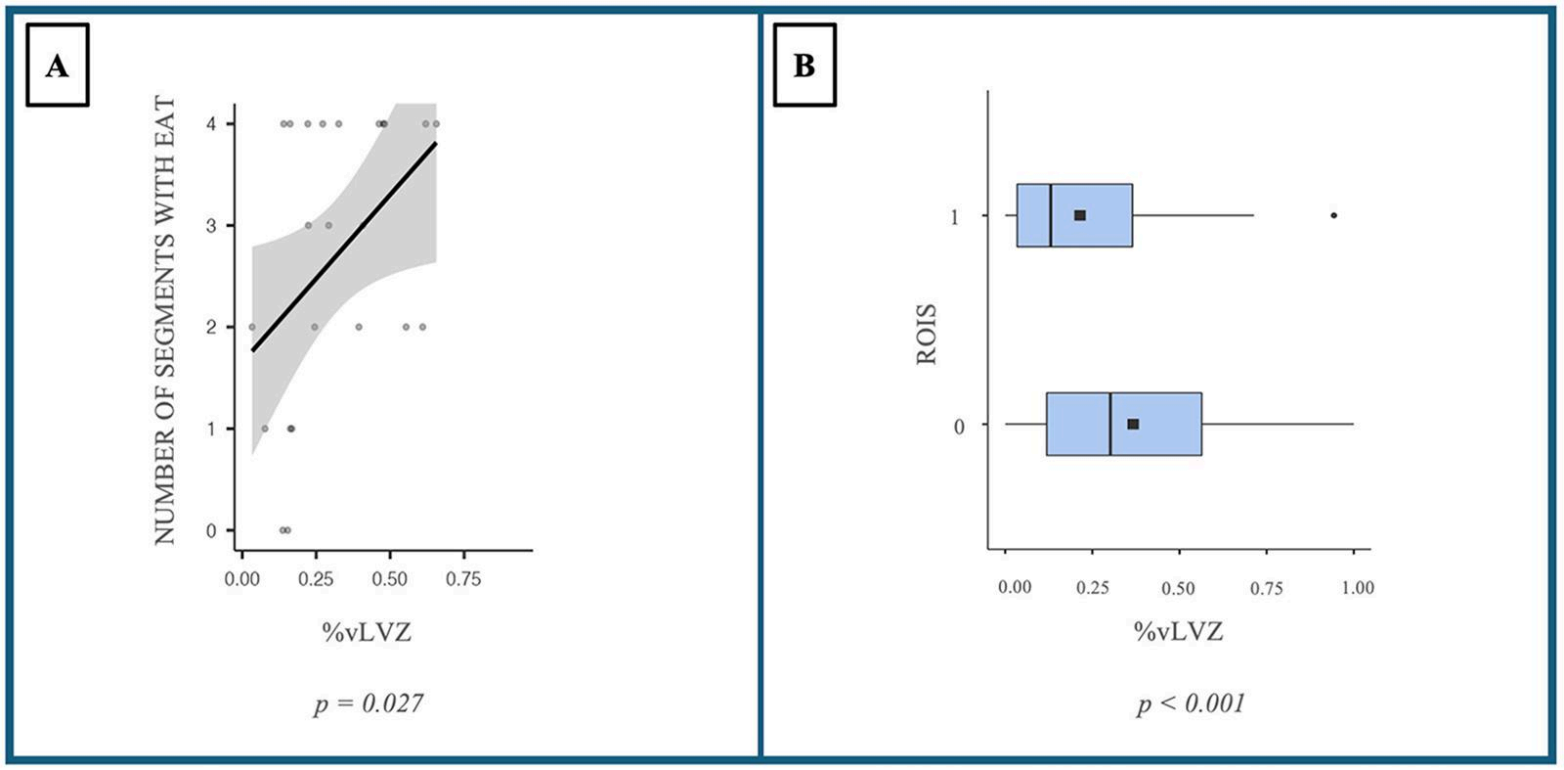
Correlation between very low voltage zones (%vLVZ) and: **(A)** epicardial adipose tissue (EAT); **(B)** regions of interest (ROIS).

A greater extent of low-voltage areas appeared to be associated with a larger presence of EAT (rho = 0.461; *p* = 0.027) (Figure 5).

Moreover, a highly significant association was observed between higher bipolar voltage and a greater prevalence of ROIs, including both rotational and focal activities (*p* < 0.001), and between a higher percentage of vLVZs and fewer ROIs and fewer focal/rotational activities (*p* < 0.001).

This relationship was confirmed when considering all atrial segments from a single patient. In fact, the presence of at least two segments with ROIs, including focal and rotational activities, was associated with higher median bipolar voltage (*p* = 0.05).

#### 3.4.3 EAT

**Figure 6:**
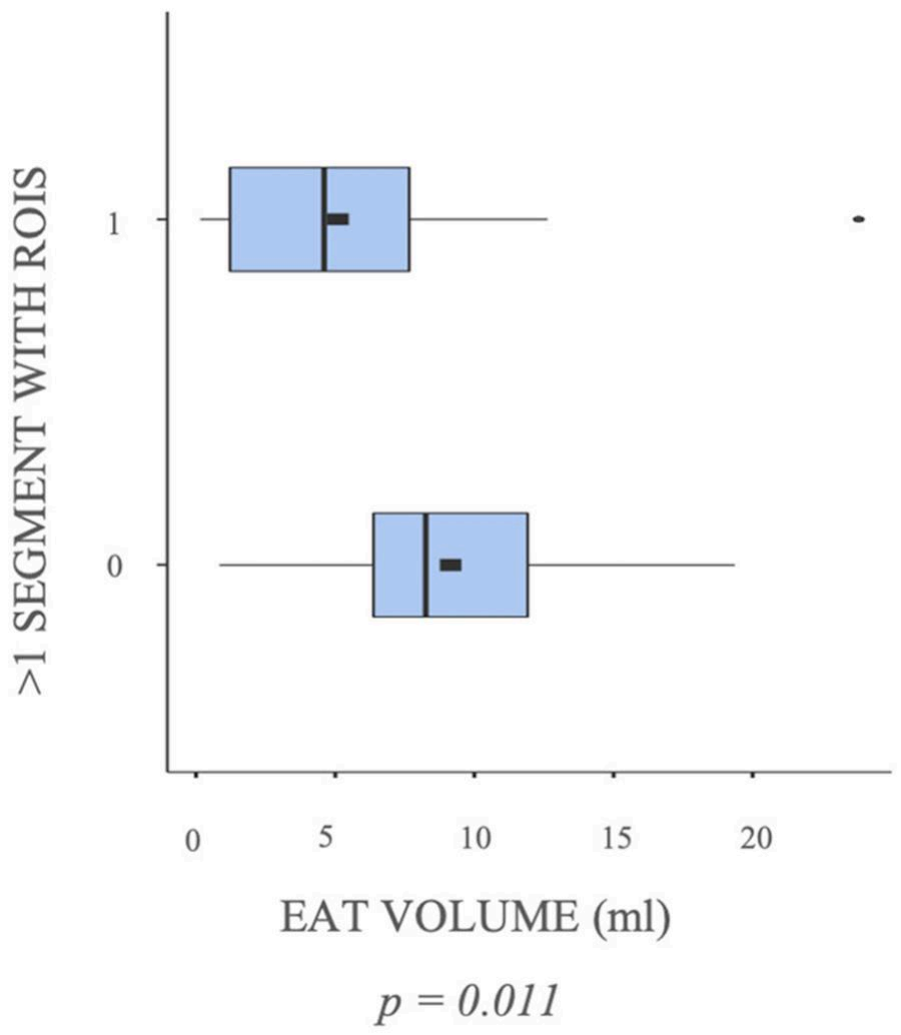
Correlation between epicardial adipose tissue (EAT) volume and regions of interest (ROIS).

The median volume of atrial EAT in our population was 5.25 mL (IQR 6.96). An inverse association was found with the presence of ROIs: to have at least two segments with ROIs or focal activities was associated with reduced EAT volume (*p* = 0.011 and *p* = 0.025, respectively) and area (*p* = 0.005 and *p* = 0.012, respectively) (Figure 6).

### 3.5 Correlation between atrial severity predictors and freedom from AF recurrences at follow-up

**Figure 7:**
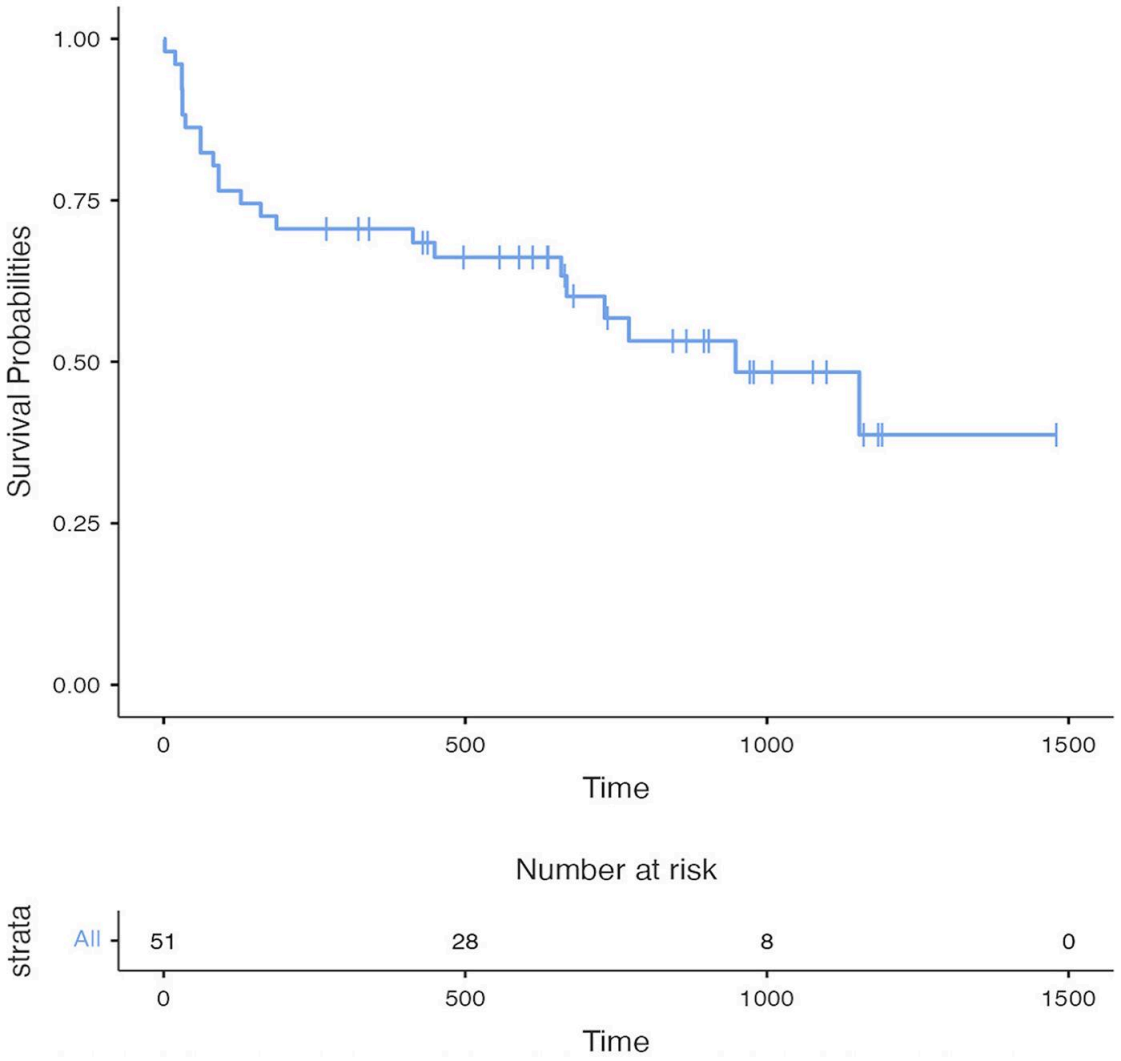
Survival curve.

Median follow-up duration was 845 days (IQR 427). Freedom from recurrence at 1 year was 70.6%. Median time to recurrence was 91 days (IQR 521) (Table 7; Figure 7).

**Table 7:** Follow-up results.

Freedom from recurrence	Total (*n* = 51)
3 months	43 (84.3%)
6 months	38 (74.5%)
12 months	36 (70.6)

In the multivariate survival analysis the mean percentage of vLVZ across atrial segments was associated with a higher risk of recurrence (HR = 1.025, CI 1.007–1.044, *p* = 0.008).

When evaluating the impact of individual segments, in our population the presence of EAT in the posterior wall appeared to be protective against recurrences (HR 0.267, CI 0.102–0.700, *p* = 0.007), while the percentage of vLVZ in the roof segment seemed to be associated with an increased risk of recurrences (HR 1.024, CI 1.003–1.045, *p* = 0.026). Finally, the presence of ROIs in the anterior wall appeared to be protective against recurrences in our population (HR 0.287, CI 0.099–0.828, *p* = 0.021).

**Figure 8:**
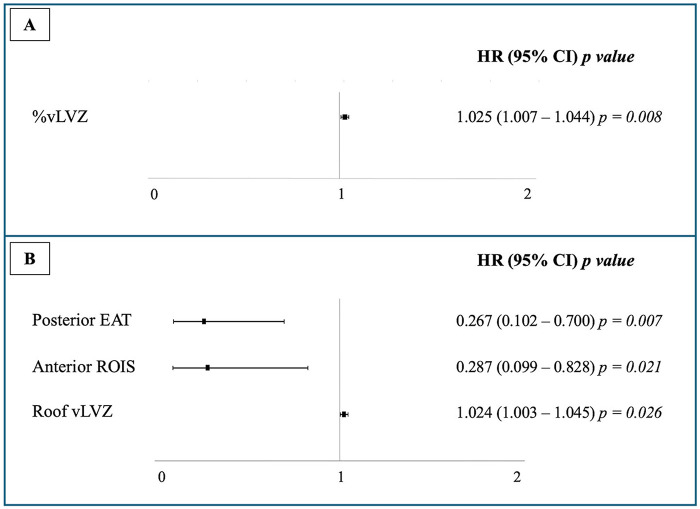
Forest plot of multivariable Cox regression analysis for arrhythmia recurrence. **(A)** Impact of mean percentage of very low-voltage zones (vLVZ) across atrial segments on recurrence risk. **(B)** Segment-specific predictors of recurrence: posterior epicardial adipose tissue (EAT), anterior regions of interest (ROIs), and roof very low-voltage zones (vLVZ).

The final multivariable Cox model showed good discriminative performance, with a Harrell’s concordance index (C-statistic) of 0.776 for predicting the time-to-event outcome (Figure 8).

## 4 Discussion

Persistent AF remains a significant challenge in the field of cardiac electrophysiology.

Over the past decade, multiple factors have been proposed as predictors of LA substrate severity. However, evidence from prior studies has been inconsistent, probably because of methodological variability, heterogeneous study populations, and because these predictors were typically evaluated in isolation rather than in an integrated fashion. In this context, the present work represents the first study to simultaneously correlate four possible substrate predictors through an integrated approach with a segment-by-segment analysis of the LA by leveraging an AI platform to support data analysis. The following section outlines and discusses the significant results observed for each selected predictor.

### 4.1 Left atrial voltage

LA bipolar endocardial voltage mapping has emerged as an invasive tool to define the AF substrate during ablation. However the methodology for defining LVZs remain non-standardized and several non-substrate factors may influence electrogram voltage, such as activation direction, electrode spacing, electrode size, tissue contact and filtering (7). Multiple studies have attempted to correlate bipolar voltage with fibrosis, a factor involved in AF initiation and maintenance (8), with conflicting results (9–11). Voltage-guided ablation, targeting LVZs, has been proposed as a patient-tailored approach, with conflicting outcomes in randomized trials (12–15).

Recently, platforms based on artificial intelligence systems have been increasingly used for handling complex datasets (16, 17). In our study, CARTONET played a crucial role. Utilizing machine learning, the platform facilitates the consultation and analysis of procedural parameters, providing the ability to retrospectively review CARTO system cases remotely and giving access to multiple layers of statistical and analytical data (18). The usefulness of CARTONET in successfully predicting possible reconnection sites following AF ablation procedures has been described (19). In our analysis CARTONET was used to segment the LA and to provide detailed analyses of voltage data for each segment. The left atrial auto-segmentation function has not been validated in the literature, but analogous features of the CARTONET platform, including the ability to automatically localize ablation tags, have demonstrated good reliability (20), with improving performance in more recent software versions (21).

Regarding the distribution of voltage alterations in our cohort, the inferior wall displayed the highest percentage of LVZs, with significant difference compared with the other segments (*p* = 0.04), while the posterior wall showed a significantly lower median bipolar voltage compared with other segments (*p* = 0.05). Conversely, the left lateral wall exhibited the lowest proportion of LVZs among all segments (*p* = 0.025) (Table 3, Figure 2).

We observed a correlation between a greater extent of LVZs and older age, and between a greater extent of LVZs and poorer renal function, findings that have already been described in the literature (22, 23).

Low voltage is the factor that, in our population, appeared to have the greatest adverse impact on recurrence-free survival probably due to the functional alterations associated, a finding consistent with literature (24, 25).

### 4.2 Left atrial wall thickness

Post-mortem studies report average measurements of LAWT between 1 and 4 mm. (26) Evidence on the relationship between AF and LAWT shows conflicting results: most studies report increased LAWT in AF (27), while others describe reduced thickness (28, 29) or dynamic changes with disease progression (30).

A higher AF recurrence rate in patients with higher LAWT values has been reported, presumably due to the lack of lesion transmurality in areas with increased wall thickness (31). Titrating PVI lesions according to local LAWT appears to be an effective strategy (32, 33).

When comparing the distribution of LAWT across segments in our cohort, the anterior wall showed the highest prevalence of zones < 1 mm thick (*p* = 0.005). Conversely, the lateral wall exhibited the highest prevalence of zones >4 mm thick, representing the segment most frequently affected by a marked increase in wall thickness (*p* = 0.005). The inferior wall demonstrated the lowest prevalence of zones <1 mm thick among all segments (*p* = 0.005) (Table 4, Figure 2B).

We identified a correlation between a greater wall thickness and a lower prevalence of low-voltage areas within the same segment. This finding is supported by published data reporting an association between reduced wall thickness and low-voltage areas (34). It is plausible that increased thickness reflects a larger amount of healthy myocardium, whereas reduced thickness may indicate areas of fibrosis. Experimental studies have shown that rapid atrial excitation during AF may lead to relative atrial myocardial ischemia and apoptosis. Elevated left atrial pressure may also be associated with wall thinning, and atrial stretch is an important fibroblast activator and stimulus for atrial fibrosis (35).

### 4.3 Epicardial adipose tissue

EAT is the fat surrounding the heart, located between the myocardium and visceral pericardium, and it has strong interactions with the underlying cardiac muscle. It is a metabolically active organ, containing autonomic ganglia, stromo-vascular structures and immune cells, providing free fatty acids and secreting numerous anti-inflammatory cytokines with protective functions on the heart in physiologic conditions (36).

However, under various pathological conditions EAT appears to be independently associated with AF incidence, severity, and recurrence (37). Possible mechanisms include direct myocardial infiltration and the release of paracrine mediators promoting inflammation, oxidative stress, fibrosis and autonomic dysfunction (36). Larger EAT volumes have been described in persistent vs. paroxysmal AF (37, 38). EAT accumulation has also been linked to post-ablation AF recurrence (38, 39).

Regarding the distribution of EAT across left atrial segments in our cohort, the inferior wall was found to contain the greatest amount of EAT and the posterior wall showed, on the opposite, the least amount of EAT. However, both correlations were not statistically significant (*p* = 0.160 and *p* = 0.200 respectively) (Table 5, Figure 2C**)**.

We found a strong correlation between epicardial fat and both BMI (*p* = 0.001) and BSA (*p* < 0.001).

In our cohort, EAT correlated with LA size: patients with more dilated atria showed a greater amount of EAT, a correlation consistent with literature (40).

Moreover, in our patients, the presence of EAT was associated with a higher prevalence of low-voltage areas. In the literature, studies have reported correlations between EAT and low-voltage areas or fibrosis, with some studies identifying regions of abnormal bipolar voltage beneath epicardial fat deposits (41), while others describe correlations without spatial co-localization (42).

A correlation was finally observed between a diffusely reduced LAWT and a lower amount of EAT in the same patient. This finding may reflect, at least in part, intramyocardial adipose infiltration (43) and may also be influenced by CT-related measurement limitations. In particular, because of the limited soft-tissue contrast of CT, the partial-volume effect can blur tissue interfaces thereby affecting boundary delineation and LAWT estimates (44).

In our population the impact of global EAT volume on the risk of AF recurrence did not seem to be significant, even when adjusting for potential confounding.

However, when assessing the effect of EAT presence in individual segments, fat in the posterior wall appeared to be protective against recurrences. This is partially in contrast with most of the available literature, but some other studies have reported similar findings: a reduced incidence of postoperative AF has been described in patients in whom cardiac fat pads were preserved during cardiac surgery (45, 46), and a protective effect of the adiponectin produced by epicardial fat has also been identified (47).

### 4.4 AF drivers

AF drivers are electrically mappable mechanisms that sustain, rather than initiate, fibrillatory conduction (48). Potential mechanisms of AF maintenance include rotational activities or the presence of foci with increased automaticity located in areas other than the PVs.

The use of CARTOFINDER in AF has shown benefits in terms of AF termination, cycle-length prolongation (49–51), and reduced recurrence (52). However, a trial on 202 patients targeting only focal activations found no improvement in arrhythmia-free survival compared with PVI alone (53).

Regarding the distribution of ROIs across segments in our cohort, the inferior and anterior walls exhibited the highest percentage of focal activity, with statistically significant differences compared with other segments (*p* = 0.005 and *p* = 0.03, respectively). The inferior and anterior walls also showed the greatest number of total ROIs, with significant differences compared with the other segments (*p* = 0.01 and *p* = 0.03, respectively). The posterior wall showed the lowest number of focal activity and ROIs overall (*p* = 0.005 and *p* = 0.012) (Table 6, Figure 2D). Literature data report that the areas with the highest number of ROIs are the left atrial appendage and the most inferior and lateral walls of the left atrium (52, 53).

With respect to the correlation between endocardial voltage and the presence of AF drivers, we found higher median voltage values in segments where AF drivers were identified. Low-voltage areas may be less likely to sustain functional re-entry, as fibrosis reduces myocyte coupling and interferes with rapid wavefront propagation (54). Several studies have investigated the correlation between AF drivers and bipolar voltage in the LA, with conflicting results: some studies found no anatomical correlation with low-voltage areas (51, 55) or fibrosis (56). Other studies reported spatial correlation between ROIs and low voltage areas (49, 57), while, in agreement with our findings, a higher LA voltage was found in sites that housed rotational activity in a study on 85 patients (58).

Regarding the correlation between drivers and LAWTs, in our cohort a greater wall thickness was associated with fewer rotational activities at CARTOFINDER analysis. This finding has also been reported in the literature: results from ex-vivo mapping found that driver regions were thinner than the rest of LA regions (59), and in a digital twins-based analysis rotational drivers tended to localise in regions with smaller LAWT (60).

Additionally, in our population, diffuse EAT was associated with a reduced number of ROIs identified by CARTOFINDER. Currently, there is no direct evidence in the literature correlating ROIs identified by the CARTOFINDER module with atrial EAT; however, in contrast with our findings, there is evidence that areas of EAT accumulation correlate with sites of high dominant frequency (61).

When assessing the prognostic effect of ROIs in individual segments, their presence in the anterior wall seemed to be protective against recurrences in our population. This result may be explained by the preferential identification of CARTOFINDER ROIs in atria with a more preserved electrical substrate, whereas ROIs are less frequently observed in atria with diffuse pathologically low voltages, which are, in turn, linked to worse outcomes. Moreover, the ablation strategy in this cohort extended beyond PVI, as additional lesions were often delivered to target the ROIs identified. Consequently, the observed protective effect may be attributable to the recognition and appropriate treatment of arrhythmogenic driver regions.

However, this potentially protective effect was observed in the anterior wall and not in other regions of the LA. A factor that distinguishes the anterior wall from other segments is the insertion of Bachmann's bundle in this region, and it is known that good interatrial conduction is a protective factor against atrial fibrillation recurrence (62). A possible explanation may be the association between repetitive focal activity in the anterior wall and marked Bachmann's bundle activity (63), which could ultimately be associated with a reduced risk of recurrence. However, dedicated studies would be required to support this hypothesis.

### 4.5 Strengths and limitations

This study has several limitations. First and foremost, the enrolled population was quite heterogeneous, with a considerable proportion of the patients having previously undergone various types of ablation procedures. In this context, it may be postulated that, particularly when assessing bipolar voltage, previous ablation lines could substantially influence the values derived from substrate analysis. It should be noted, however, that in the vast majority of our cases prior ablation was limited to PVI, and the PVs were excluded from voltage analysis using CARTONET.

Another limitation is that the current version of CARTONET does not allow direct integration with the ADAS platform; consequently, although left atrial segmentation for voltage data was performed automatically using the AI-based platform, allocation of the remaining variables across atrial segments was performed manually by three experienced operators using CARTONET-defined boundaries as anatomical references.

Moreover, since to be included in the study patients had to remain in atrial fibrillation after PVI so that the CARTOFINDER algorithm could be launched, bipolar voltage data were acquired in AF, in contrast to most of the literature on atrial low-voltage areas. In addition, cutoffs of 0.2 and 0.5 mV were used, as these are the cutoffs automatically provided by the CARTONET platform, and these values are not customizable in the current version of the platform. Nevertheless, the literature is not unanimous regarding the most appropriate methods for measuring endocardial voltage and the cutoff values to be used.

The analysis of the impact of predictors on recurrence rates is limited by the fact that the ablation procedures from which the data were collected were not restricted to PVI, since in most cases additional ablation lines were delivered according to a patient-tailored strategy. Although additional ablation lines were accounted for in the multivariable analysis, heterogeneity in procedural strategies may still obscure the true effect of the predictors.

In segment-level analyses, observations from the same patient were treated as independent, without accounting for within-patient clustering. This may have led to an underestimation of variability and inflation of statistical significance.

Additionally, although missing data were handled using imputation techniques, complete-case sensitivity analyses were not performed. Restricting the analysis to complete cases would have resulted in a substantial reduction in the number of events (from 23 to 15), significantly limiting statistical power and model stability. Therefore, imputation-based analyses were considered the most appropriate approach in this setting.

On the other hand, a notable strength of this study is that for follow-up data collection we relied in a substantial proportion of cases on the analysis of device-recorded events, gaining access to an accurate assessment of even asymptomatic recurrences.

An additional innovative element of this study lies in the use of CARTONET, a platform that leverages artificial intelligence to provide advanced automated analysis.

Given the retrospective nature of this study, our aim is not to propose criteria for performing additional ablation lines in the LA. Rather, this work represents a preliminary, exploratory analysis that requires evidence from larger, randomized studies to shed light on the true clinical significance of the observed alterations.

## 5 Conclusion

While the need to tailor ablation procedures to the individual patient's atrial substrate has become increasingly evident, efforts to identify reliable predictors of LA substrate abnormalities have so far resulted in limited or inconclusive findings. Our multidimensional and segment-by-segment assessment of the LA suggested several correlations between the four predictors of atrial substrate severity, providing new insights into how structural and functional alterations interact in shaping atrial remodeling. Understanding how factors associated with atrial substrate abnormalities interrelate may help tailor the procedure more closely to the individual patient. At follow-up, the presence of widespread low-voltage areas across LA segments seemed to be the predominant predictor of post-ablation recurrence, while also other segment-specific predictors appeared to have an impact. The use of AI-based platforms highlights the potential of advanced computational systems to manage complex information. From our analysis the capabilities of the CARTONET platform are evident, readily providing analyses and insights on previously acquired maps that would otherwise be accessible only with intricate and time-consuming manual extrapolations. A wise employment of these technologies, integrating advanced imaging tools and high-density electroanatomical mapping, may substantially advance our understanding of the pathophysiological mechanisms underlying AF and improve the optimization of ablative treatments.

## Data Availability

The datasets presented in this article are not readily available because datasets not publicly available due to ethical and privacy restrictions related to patients data. Requests to access the datasets should be directed to iacopino@iol.it.
