# Supplementary material for: Segmental analysis of left atrial substrate severity predictors in patients undergoing persistent atrial fibrillation ablation
Source: Front Cardiovasc Med. 2026 Jul 6;13:1830208. doi: 10.3389/fcvm.2026.1830208 (PMC13381624; doi:10.3389/fcvm.2026.1830208)
Supplement: Supplementary file 1 [file Table1.docx]

**Follow-up data**. CIED: Cardiac Implantable Electronic Device.

| **TYPE OF FOLLOW-UP** | **TOTAL (N = 69)** |
| --- | --- |
| CIED telemonitoring | 8 (11%) |
| Telephone call | 10 (14%) |
| Outpatient visit | 25 (36%) |
| Hospital readmission | 8 (12%) |
| No Follow-Up | 18 (26%) |

**Correlation between atrial substrate severity predictors and anthropometric - biochemical parameters**

Correlations between EAT and BSA/BMI.


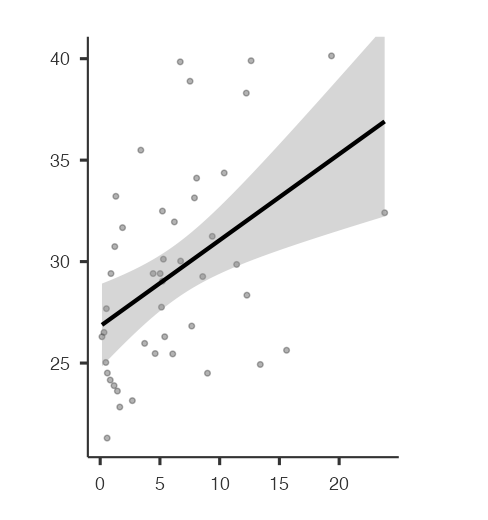


BMI

EAT VOLUME (ml)

rho = 0.543

p = 0.004


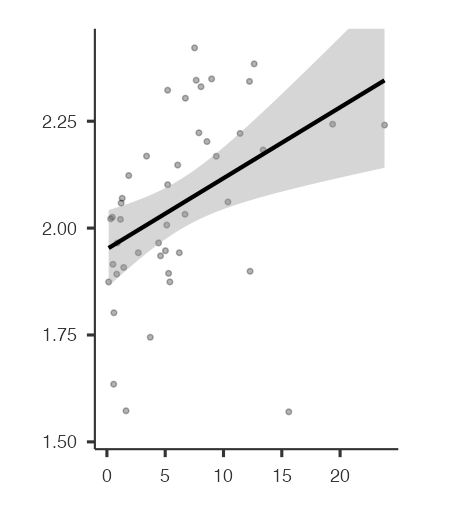


BSA

EAT VOLUME (ml)

rho = 0.543

p = 0.004


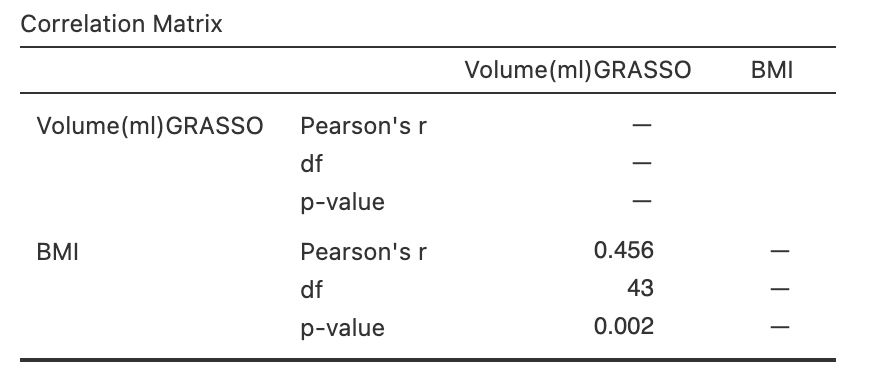
Correlations between EAT and LA dimensions:

SEGMENTS WITH EAT

LA AREA (cm2)


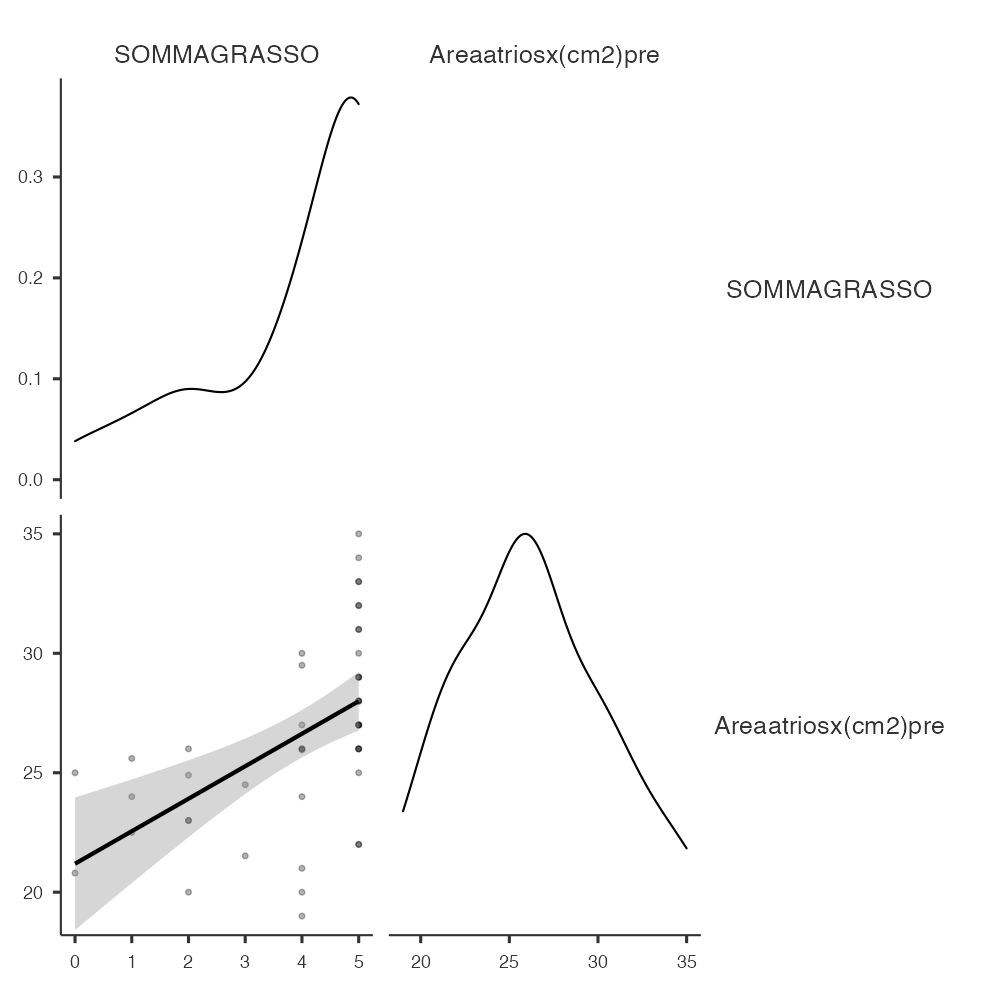


rho = 0.643

p = <0.001

LA VOLUME (mL)


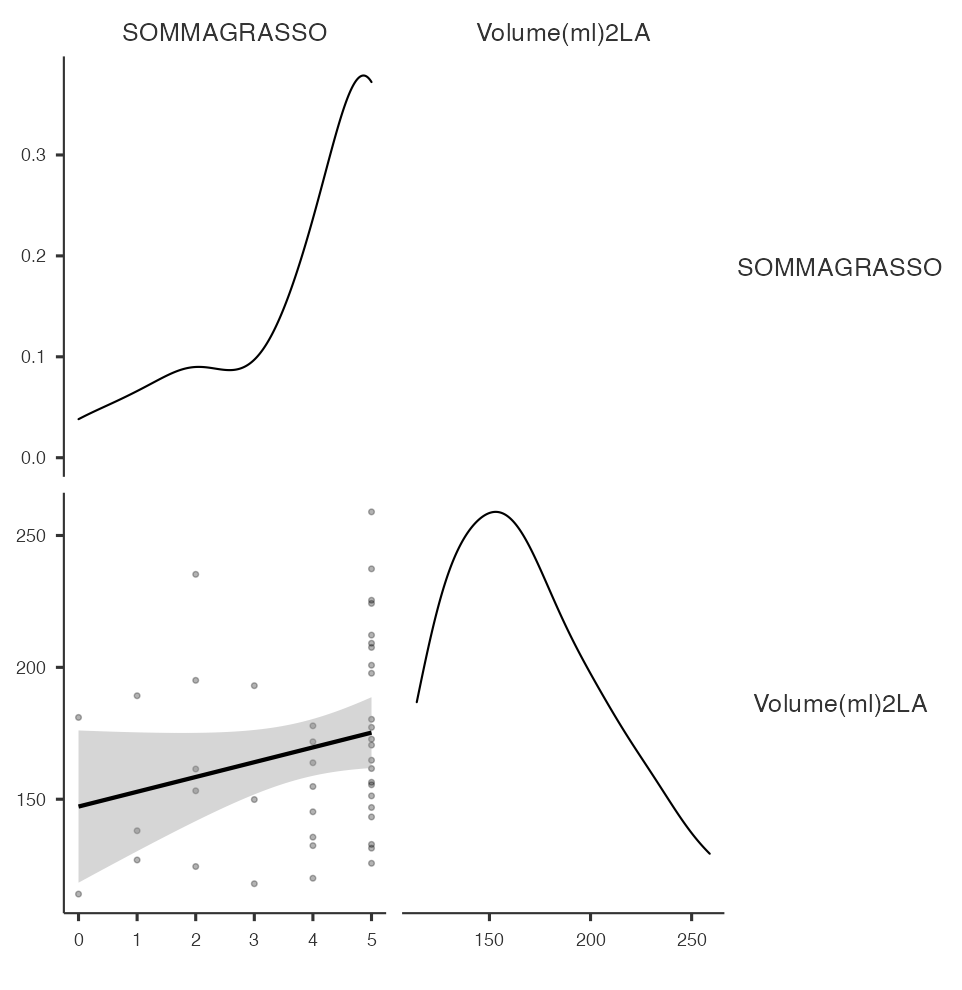


SEGMENTS WITH EAT

rho = 0.298

p = 0.049

Correlations between low-voltage areas and glomerular filtration rate (GFR).


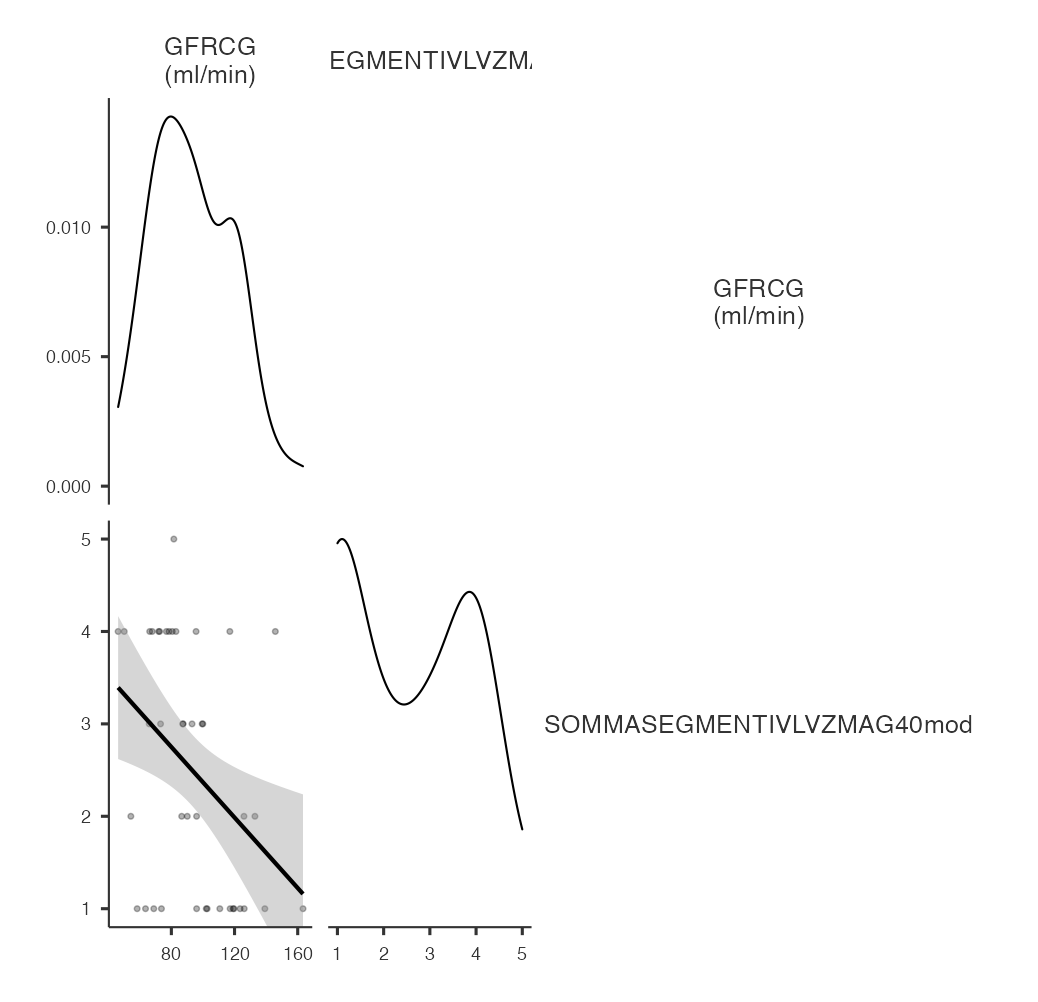


SEGMENTS WITH vLVL >40%

GFR (ml/min)

rho = -0.404

p = 0.08

Correlation between low-voltage areas and age.


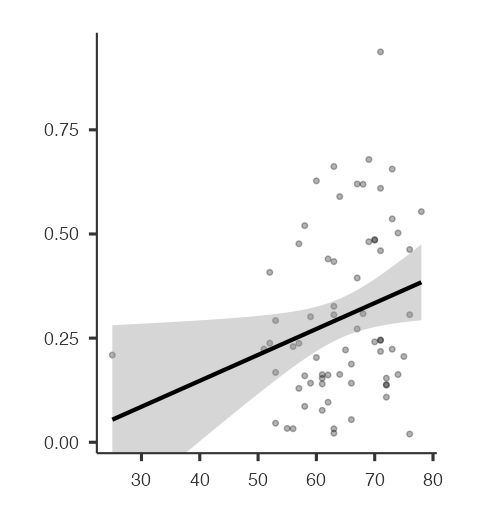


r = 0.26

p = 0.031

AGE

MEAN % vLVZ

**Correlations between bipolar voltage and ROIs:**


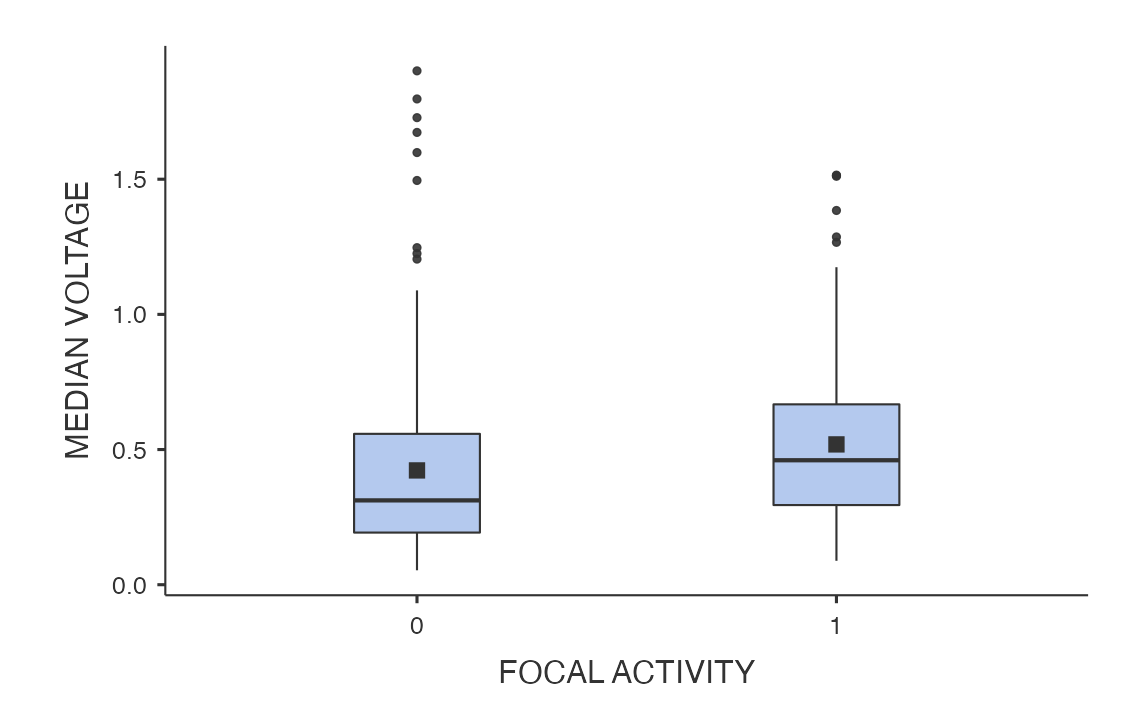


*p <0.001*


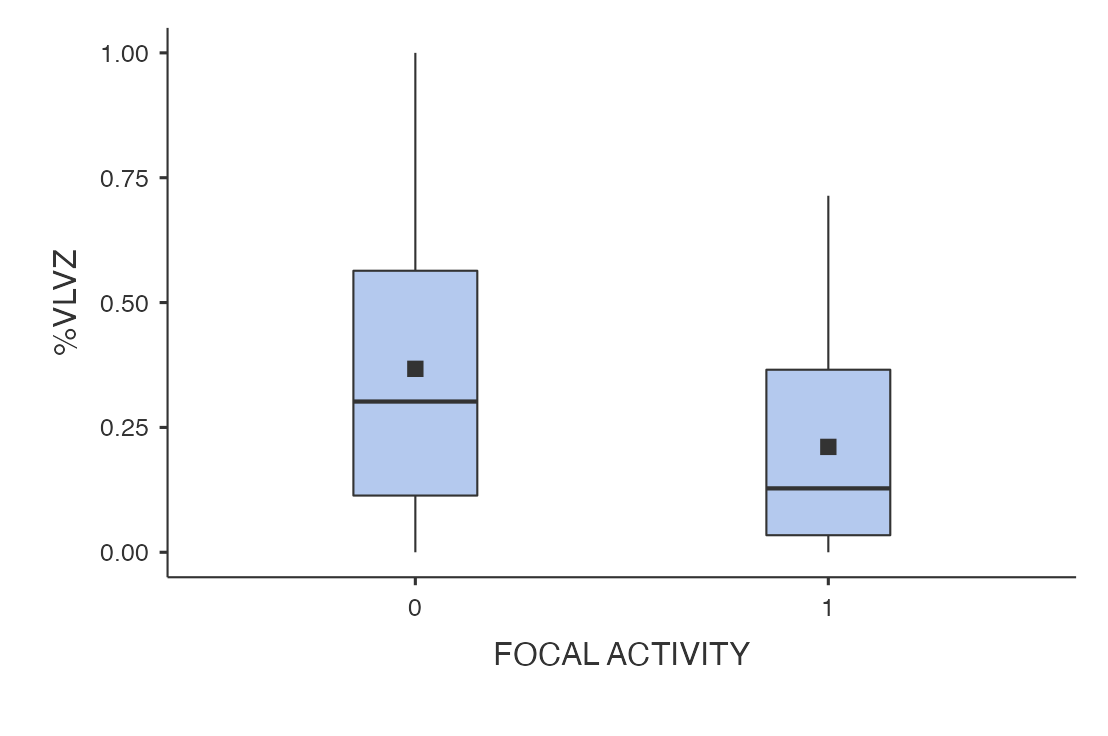


*p <0.001*


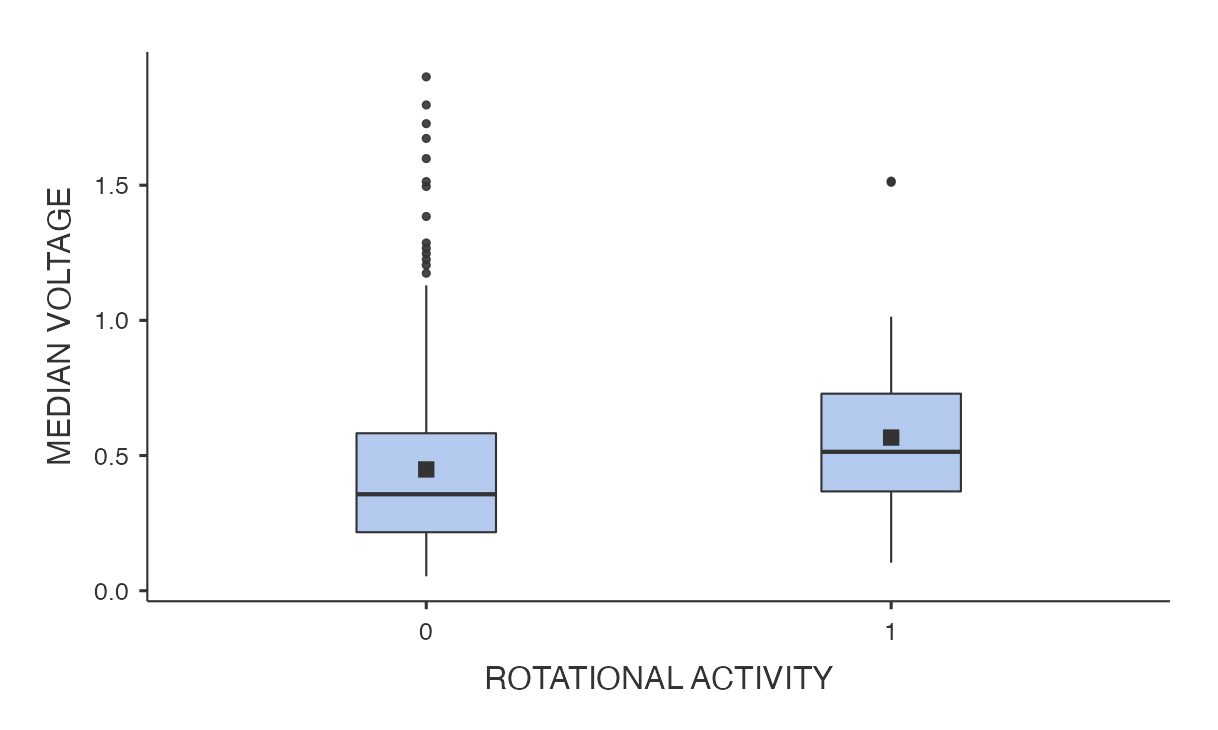


*p <0.001*


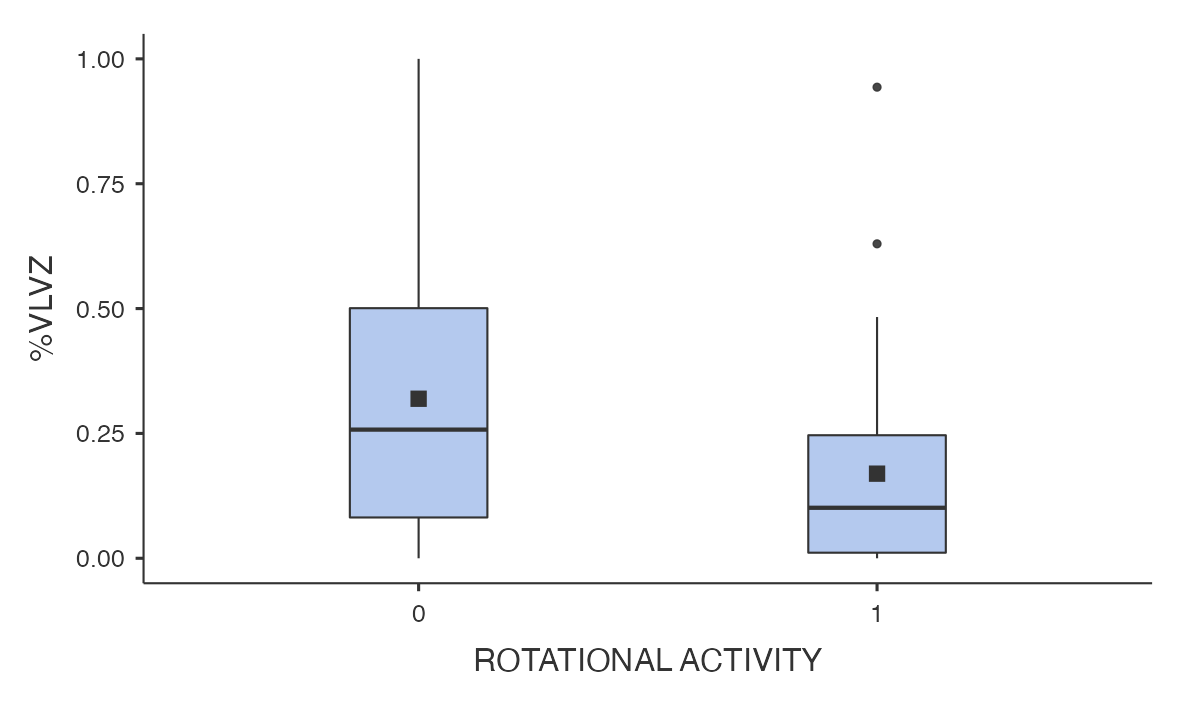


*p <0.001*


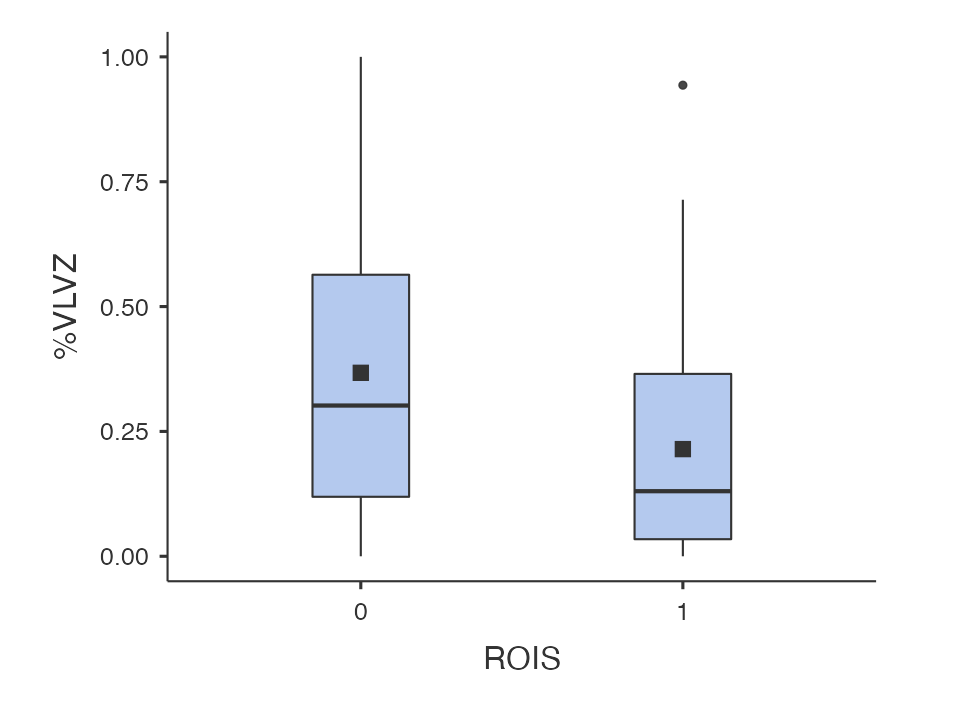


*p <0.001*


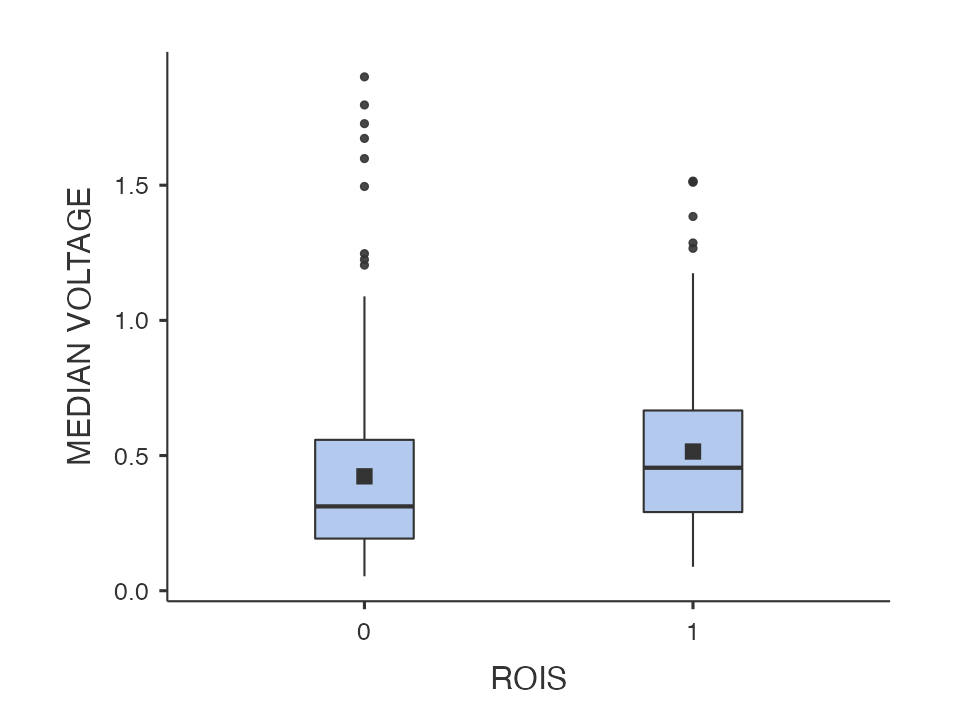


*p <0.001*

**Further correlations between LAWT and ROIs**: in the presence of thinner walls, rotational activities exhibited a shorter cycle length (CL) and lower maximum repetitiveness activity (MAX REP) (p=0.006 and p=0.005, respectively).


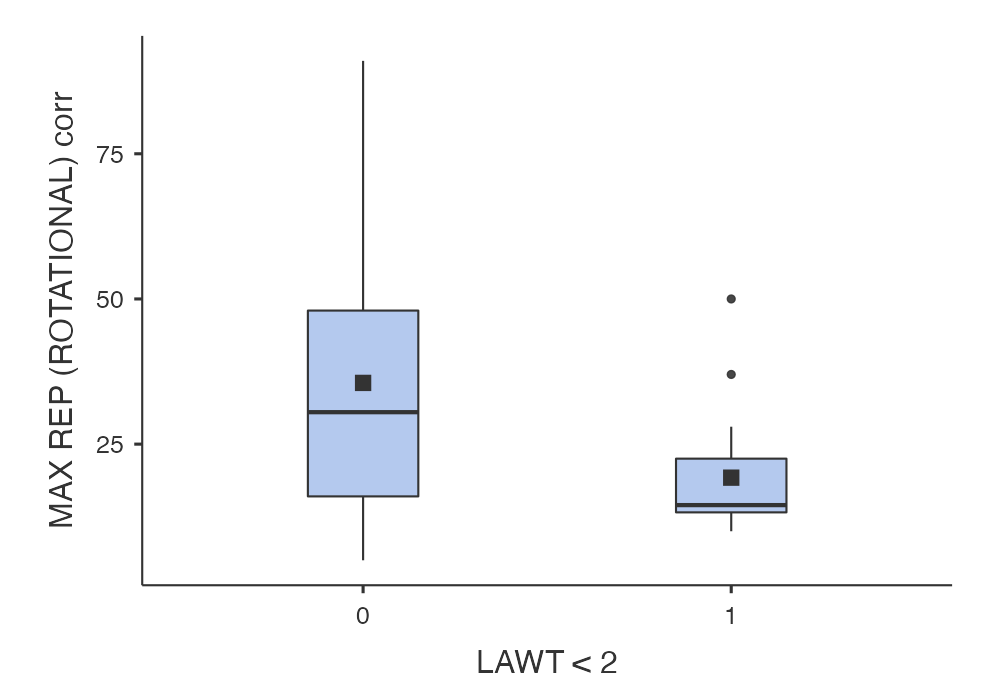

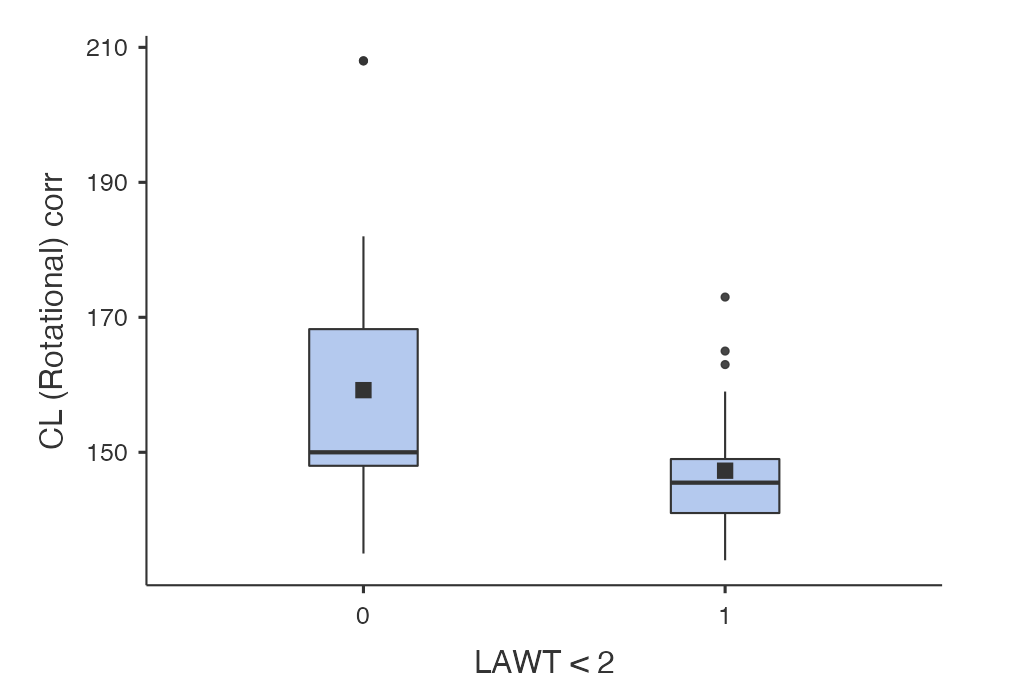


*p = 0.006*

*p = 0.005*

ROTATIONAL ACTIVITY CL

ROTATIONAL ACTIVITY MaxRep

ROTATIONAL ACTIVITY MAX REP

ROTATIONAL ACTIVITY CL

**Survival analysis**

We observed no significant difference in freedom from recurrence between patients who had previously undergone an ablation procedure and ablation-naïve patients.


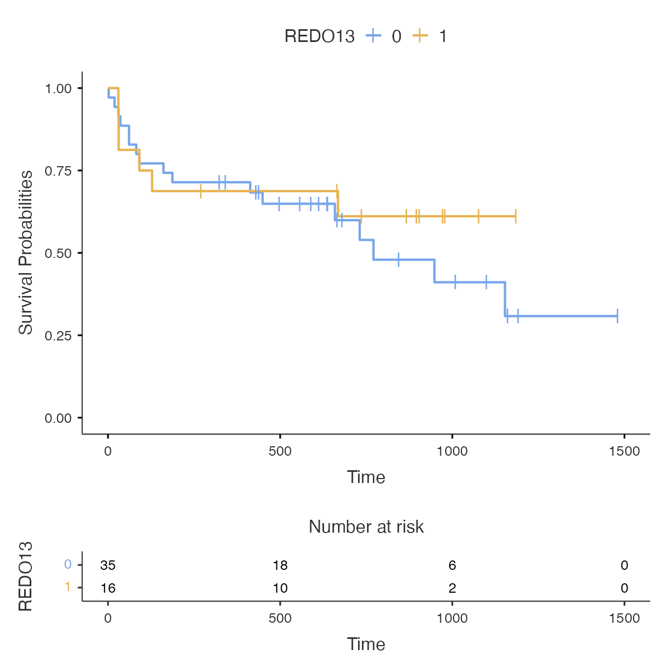


*p = 0.470*

REDO

REDO

Moreover, no difference in survival was found according to the type of ablation catheter used.


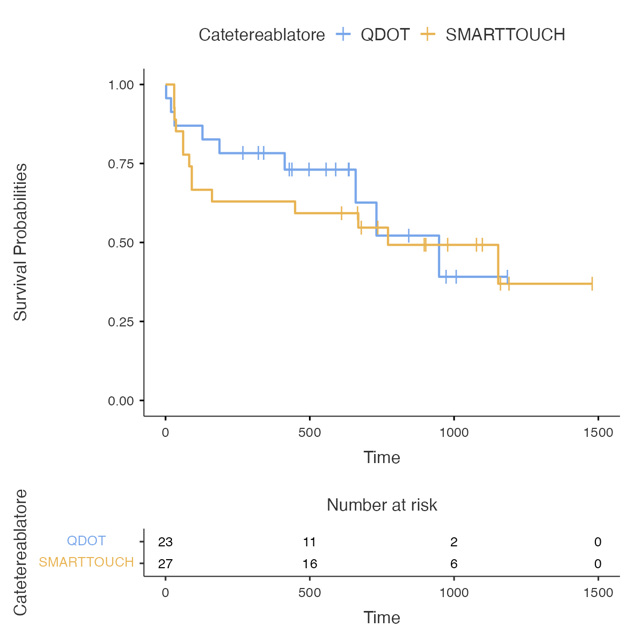


CATHETER

CATHETER

ST SF

QDOT

CATHETER


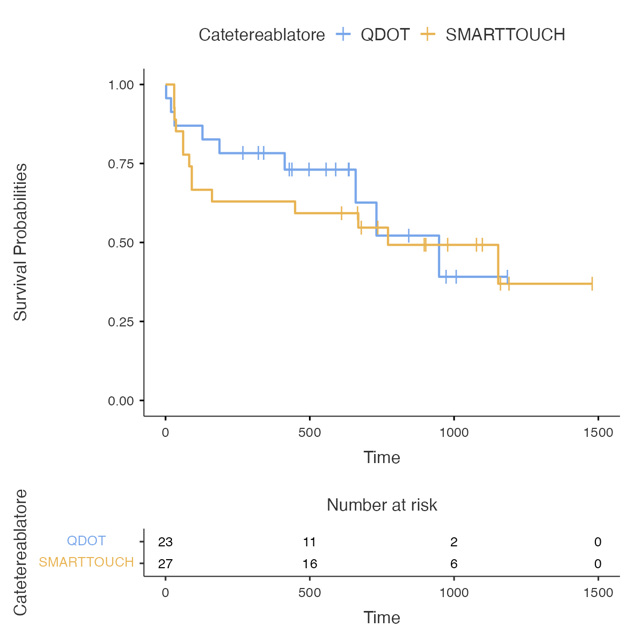


ST SF

*p = 0.699*
